# INCLUSION OF SPIRITUAL CARE AT THE INDIVIDUAL LEVEL FOR PERSONS LIVING WITH DEMENTIA

**DOI:** 10.1093/geroni/igae098.2552

**Published:** 2024-12-31

**Authors:** Sooyoung Park, Eunkung Kim, Soobin Park

**Affiliations:** Samsung Medical Center, Seoul, Republic of Korea; Midwest University, St. Louis, Missouri, United States; Washington University in St. Louis, St. Louis, Missouri, United States

## Abstract

Spirituality is known to remain for a long time until the advanced stages of dementia and is associated with positive health outcomes. Initiatives such as dementia-friendly church services and spiritual programs in institutional settings aim to recognize and enhance the emotional, social, spiritual, and artistic facets of person living with dementia (PLWD). However, the application of spirituality in the day-to-day lives of PLWD at home remains a research gap and poses practical challenges. Thus, we draw from our personal insights as a family caregiver of an older adult with severe dementia and collaborated with a social worker specialized in this area to integrate spirituality into PLWD’s daily routines. Based on the Spiritual Dimension of Ageing model, we designed a range of practices aimed at fostering sustainable spirituality at home for PLWD. These practices encompassed four themes: relationships with others, creating an environment conducive to engaging in meaningful activities, incorporating favorite art/music, and exploring religious beliefs. This approach allowed for the customization and diversification of spirituality practices, catering to the unique interpretations and needs of each PLWD. As part of the practice, it enabled PLWD to reunite with a family member after 12 years. Utilizing photographs and written narratives from community-dwelling PLWD and caregivers, we highlighted specific qualitative benefits of this approach (e.g., positive emotions, experience moments “beyond dementia”), particularly for individuals in the advanced stages of dementia. Discussing opportunities to nurture the whole person and their soul may offer comfort and hope to those facing a disease with no cure.

